# Gut Microbiota in Systemic Lupus Erythematosus and Correlation With Diet and Clinical Manifestations

**DOI:** 10.3389/fmed.2022.915179

**Published:** 2022-06-30

**Authors:** Xiao Wang, Qiang Shu, Lijun Song, Qi Liu, Xiaoxia Qu, Ming Li

**Affiliations:** ^1^Department of Rheumatology, Qilu Hospital of Shandong University, Jinan, China; ^2^Shandong Province Clinical Research Center for Immune Diseases and Gout, Jinan, China; ^3^Department of Gastroenterology, Qilu Hospital of Shandong University, Jinan, China

**Keywords:** Gut microbiota, Systemic lupus erythematosus, Diets, *Streptococcus*, Network analysis

## Abstract

Despite the existing studies relating systemic lupus erythematosus (SLE) to changes in gut microbiota, the latter is affected by external factors such as diet and living environment. Herein, we compared the diversity and composition of gut microbiota in SLE patients and in their healthy family members who share the same household, to link gut microbiota, diet and SLE clinical manifestations. The study cohort included 19 patients with SLE and 19 of their healthy family members. Daily nutrition was assessed using a food frequency questionnaire (FFQ). Microbiota was analyzed using amplicons from the V4 regions of the 16S rRNA gene, to obtain microbiota diversity, taxa relative abundances and network analysis. The gut microbiota in the SLE group had lower alpha diversity and higher heterogeneity than the control group. SLE patients had decreased Acidobacteria, Gemmatimonadetes, Nitrospirae and Planctomycetes at the phylum level, and increased *Streptococcus, Veillonella, Clostridium_XI*, and *Rothia* at the genus level. *Streptococcus* was extremely enriched among patients with lupus nephritis. *Lactobacillus, Clostridium_XlVa, Lachnospiracea_incertae_sedis* and *Parasutterella* OTUs were associated with diet and clinical features of SLE. Finally, the gut microbiota of SLE patients remained different from that in healthy controls even after accounting for living conditions and diet.

## Introduction

The largest microbial ecosystem in humans is located in the intestinal tract, and is formed by the intestinal flora and its metabolites (1). Indeed, the number of genes encoded from gut microbes, referred to as the “second genome,” is 150 times larger than in the human genome (2). Growing evidence in the past decade suggests that gut microbe changes are involved in autoimmune diseases such as inflammatory bowel disease (IBD) (3), rheumatoid arthritis (RA) (4) or Systemic lupus erythematosus (SLE), a heterogeneous autoimmune disease characterized by overexpression of auto-antibodies that results in multi-organ injury (5, 6). However, the gut microbial community in individuals was affected by diet and household environment, among other factors. For example, both animal and human studies have shown that rapid and long-term changes in the structure and function of the gut microbiota are strongly linked to dietary patterns (7, 8). Also, individuals who share a household present similar microbiome and this accounts for 20% of the variance in microbiome β diversity due to environmental factors (9).

A possible link has been suggested between SLE and the alteration of gut microbiota, although these studies compared SLE patients with randomly selected healthy individuals, which ignored the effect of environmental factors on the gut microbiome (5, 10). Moreover, some of these studies included patients in SLE remission, which may not fully reflect the relationship between gut microbiota and disease. To address these limitations, the present study only included patients that need hospitalization treatment. Also, to account for differences in living conditions, the control group consisted of healthy family members who lived in the same household as the SLE patient. Differences in gut microbiome composition between SLE patients and control group were determined with 16S rRNA gene sequencing of fecal samples, whereas dietary patterns were obtained with a food frequency questionnaire (FFQ). With these data, we obtained a relationship between gut microbiota, dietary factors and clinical manifestations of SLE.

## Materials and Methods

### Ethics Statement

The study protocol was approved by the Ethics Committee of Qilu Hospital of Shandong University (approval number KYLL-2017–056). All participants signed a written informed consent form.

### Study Cohort and Fecal Sampling

The SLE cohort (SLE) included 19 patients admitted to the Qilu Hospital for treatment of SLE, between July 2019 and April 2020. All these patients fulfilled the American College of Rheumatology Criteria for the diagnosis of SLE (11). The healthy control (HC) group included 19 matched family members (7 parents, 9 spouses and 3 children) that lived in the same household as the SLE patients. Fresh fecal samples were collected from participants in both groups on the first day of their hospital admission and immediately stored at −80°C. None of the participants in both groups received antibiotic or probiotic treatment within 4 weeks before enrollment into the study. Also, patients in the SLE group had no other diseases, whereas participants in the HC group were disease free. None of the participants smoked or had special dietary habits such as alcohol consumption or vegetarianism. In the SLE group, patients' clinical manifestations and medication records at the time of enrollment were registered, e.g., SLE disease activity index (SLEDAI), organ injuries, and also immunological data such as antinuclear antibodies (ANAs), antiphospholipid antibodies (ACLs) and complement level.

### Nutritional Assessment

A FFQ was administered to all participants to assess their dietary intake patterns over the previous year. The FFQ was based on previous studies of dietary patterns among Chinese population and included 25 major food categories and 97 food items (12, 13). The frequency of intake for the items in the different food categories was quantified according to 1–9 scale: **1**, never eat; **2**, consumed less than once a month; **3**, consumed 1-3 times per month; **4**, 1-2 times per week; **5**, 3–4 times per week; **6**, 5–6 times per week; **7**, once daily; **8**, twice daily; **9**, ≥3 times daily. Each of these levels was assigned an increasing relative weight: 0, 0.03, 0.07, 0.22, 0.50, 0.79, 1, 2 and 3 for levels **1** through **9**, respectively. The food intake amount was quantified with six different serving sizes to choose from: ≤ 50 g, 100 g, 150 g, 200 g, ≥250 g and “not applicable”. Data collected from the FFQ was transformed into a daily energy and nutrient intake using the China Food Composition Table (14). The table indicated an average value of energy and nutrient content of each food category that make up by 3–8 individual food items. In this study, the total energy and 15 kinds of nutrients, including 6 macro and 9 trace nutrients were chosen to be analyzed. The intake was calculated as follows:


$$\begin{array}{l} Energy\;intake=\overset{i}{\underset{n=i}{\sum }}\left(f1i\;*\;f2i\;*\;Ei\right),\\ Nutrient\;intake=\overset{i}{\underset{n=i}{\sum }}\left(f1i\;*\;f2i\;*\;Ni\right) \end{array}$$

*f* 1: food frequency relative weight, *f* 2: food intake weight, E: energy content and N: nutrient content; i = 1–25 (25 categories of food in total).

Since nutrient intake is associated with the total energy of the individual, for each participant, daily nutrient was adjusted to their daily total energy intake for analysis based on the literatures (15, 16).

### 16S-rRNA Sequencing and Microbiota Profiling

All fecal samples were transferred to Novogene (Tianjin, China) for processing and analysis. Total bacterial DNA was extracted with the DNA extraction kit (DP328, Tiangen Company, Beijing, China). The V4 region of the 16S ribosomal subunit gene was amplified using the 515F and 806R barcoded paired primers and sequenced using the Illumina Nova 6000 platform as previous described (17). Valid sequences were downloaded for microbiota profiling. All valid sequencing data were prepared using Usearch (version 10.0.240) software. Briefly, valid sequences were dereplicated using the Usearch fastx_uniques algorithm. Using the Usearch cluster_otus algorithm, dereplicated sequences were clustered to the same OTU if their distance was < 0.03. The representative sequence of each OTU was then aligned to the RDP (Release 11.5) using the Sintax algorithm, with a parameter sintax_cut off of 0.8. The OTU abundances were merged at the phylum and genus levels using the sintax_summary algorithm with parameters -rank p and -rank g, respectively. The alpha and beta diversities were calculated using Usearch -alpha_div, -cluster_agg, and -beta_div algorithms.

### Statistical Analysis

The paired *t*-test was used for the comparation of demographic and food consumption data between SLE and HC group. All statistical analyses about gut microbiota were performed in R-studio (R, v.1.3.959), which is an integrated development environment for R (v.3.6.3). For clustering analysis, we first used the vegdist function of the vegan package to calculate the Bray distance of the microbiome, followed by a hierarchical cluster analysis using the “ward.D” algorithms in the hclust function. To compare the microbiota homogeneity between SLE and control groups, we used the Bray-distance-based betadisper function of the vegan package to implement Marti Anderson's PERMDISP2 procedure. This was done to analyze multivariate homogeneity of group dispersions, and was followed by an analysis of variance (ANOVA) for dimension-reduced variances (18). The Wilcoxon test was used to numerically compare the two groups. The OTU abundance, taxonomy profile, diversity profile and metadata were all tested in R. Between-group comparison of the phylum- and genus-level microbiome abundance was evaluated using the Kruskal-Wallis test. The resulting *p*-values were adjusted using the “false discovery rate (FDR)” method of the p.adjust function in R. A volcano plot was constructed to show significant between-group differences. Host–microbiota interactions were visualized by construction of a co-occurrence network of the top 50 most abundant OTUs and dietary elements. The pairwise Spearman correlation among the OTU abundances was calculated using a different cor.test function in R. For the pairwise relationship between the OTUs, a *p*-value threshold <0.01 was applied to filter significant correlations. In the patient group, the relationship between OTUs, dietary patterns and clinical manifestations of SLE was calculated and filtered by a *p*-value threshold of 0.05. All these filtered *p*-values were adjusted using the “Benjamini and Yekutieli (BY)” method of the p.adjust algorithm in R. Only correlations with an adjusted *p*-value <0.05 (q < 0.05) were exported to Cytoscape (v.3.6.1), where the network plot was constructed.

## Results

### Demographic, Clinical and Diet Characteristics of SLE and HC Groups

All participants were of Asian ethnicity and lived in the Shandong Province, China. The distribution of age, body mass index (BMI), total energy intake, macronutrients, minerals and vitamins was comparable between the two groups (Table 1). The clinical and immunological features of SLE group was listed in Table 2. Of the 19 patients in the SLE group, 12 (63.16%) were newly diagnosed with SLE, whereas 7 (36.84%) were experiencing a disease flare-up.

**Table 1:** General characteristics and mean dietary intake of SLE patients and HC.

	**SLE**	**HC**	**P–value**
N	19	19	
Age (yr)	36.11 ± 12.19	39.32 ± 10.96	0.47
BMI	21.76 ± 3.90	23.43 ± 2.32	0.13
Female, n (%)	16 (84.21)	7 (36.84)	0.003*
Total energy (kal/d)	1828.90 ± 289.18	2090.57 ± 581.38	0.08
Protein (g/d)	74.35 ± 12.75	85.23 ± 32.98	0.18
Carbohydrate (g/d)	273.99 ± 56.87	291.32 ± 91.21	0.41
Fat (g/d)	49.02 ± 13.50	61.72 ± 35.15	0.13
PUFA (g/d)	9.07 ± 2.01	11.84 ± 5.91	0.08
Dietary fiber (g/d)	14.67 ± 4.08	14.39 ± 6.43	0.82
Cholesterol (mg/d)	129.62 ± 71.86	169.42 ± 143.06	0.21
Vitamin A (mg/d)	670.50 ± 264.49	692.90 ± 458.23	0.79
Vitamin B1 (mg/d)	0.95 ± 0.22	1.09 ± 0.50	0.26
Vitamin B2 (mg/d)	1.03 ± 0.30	1.07 ± 0.45	0.74
Vitamin C (mg/d)	98.79 ± 40.02	91.64 ± 42.93	0.34
Vitamin E (mg/d)	17.18 ± 4.77	18.57 ± 8.70	0.52
Calcium (mg/d)	540.66 ± 195.14	507.70 ± 218.01	0.62
Iron (mg/d)	17.75 ± 3.62	19.01 ± 6.28	0.45
Zinc (mg/d)	10.04 ± 2.22	11.66 ± 4.51	0.14
Manganese (mg/d)	3.93 ± 1.07	4.74 ± 1.52	0.06

*Values shown are mean ± SD, unless otherwise noted*.

*BMI, Body Mass Index; PUFA, Polyunsaturated Fatty Acid*.

**Table 2:** Clinical and immunological features of SLE patients.

**Subject No**.	**Disease duration (m)**	**SLEDAI**	**Anti-dsDNA (IU/mL)**	**Clinical manifestations**	**Treatment strategies**
SLE 1	7	5	8.93	LN, FE	GCs+HCQ
SLE 2	24	9	899.50	LN, AR, FE	GCs+MMF
SLE 3	60	5	1019.81	HD, MR	GCs+HCQ+TAC
SLE 4	24	3	165.85	HD, AL	GCs+HCQ
SLE 5	2	5	9.76	MR, FE, RP	-
SLE 6	3	3	604.00	MR, FE	-
SLE 7	12	8	41.85	LN, AR,	GCs
SLE 8	0.3	5	109.39	LN, HD, MR	-
SLE 9	0.25	9	181.00	LN, HD	-
SLE 10	24	8	21.40	AR, SE	NSAIDs
SLE 11	120	4	422.55	HD, SE, RP	GCs+HCQ+MMF+RTX
SLE 12	240	5	1149.50	LN, HD	GCs+HCQ+CTX
SLE 13	12	4	69.00	AR	GCs+MTX
SLE 14	24	1	69.90	HD	GCs+HCQ+MMF
SLE 15	0.5	7	1032.04	HD, AL, SE	GCs+TAC
SLE 16	120	10	293.00	LN, NPSLE, AL, OU	GCs+HCQ+CTX
SLE 17	1	7	132.00	HD, AL	-
SLE 18	2	4	26.22	AR, MR	NSAIDs
SLE 19	0.16	3	338.11	HD, OU	-
Median (range)^a^	12 (0.16, 240)	5 (1, 10)	181 (8.93, 1149.50)	-	-

^a^*Data shown as median (minimum, maximum). AL, alopecia; AR, arthritis; CTX, cyclophosphamide; FE, fever; GCs, glucocorticoids; HCQ, hydroxychloroquine; HD, hematological disorder; LN, lupus nephritis; MMF, mycophenolate mofetil; MR, malar rash; NPSLE, neuropsychiatric SLE; NSAIDs, nonsteroidal anti-inflammatory drugs; OU, oral ulcers; RP, Raynaud's phenomenon; RTX, rituximab; SE, serositis; TAC, tacrolimus*.

### Alpha Diversity of the Microbiota in the SLE Group

**Figure 1:**
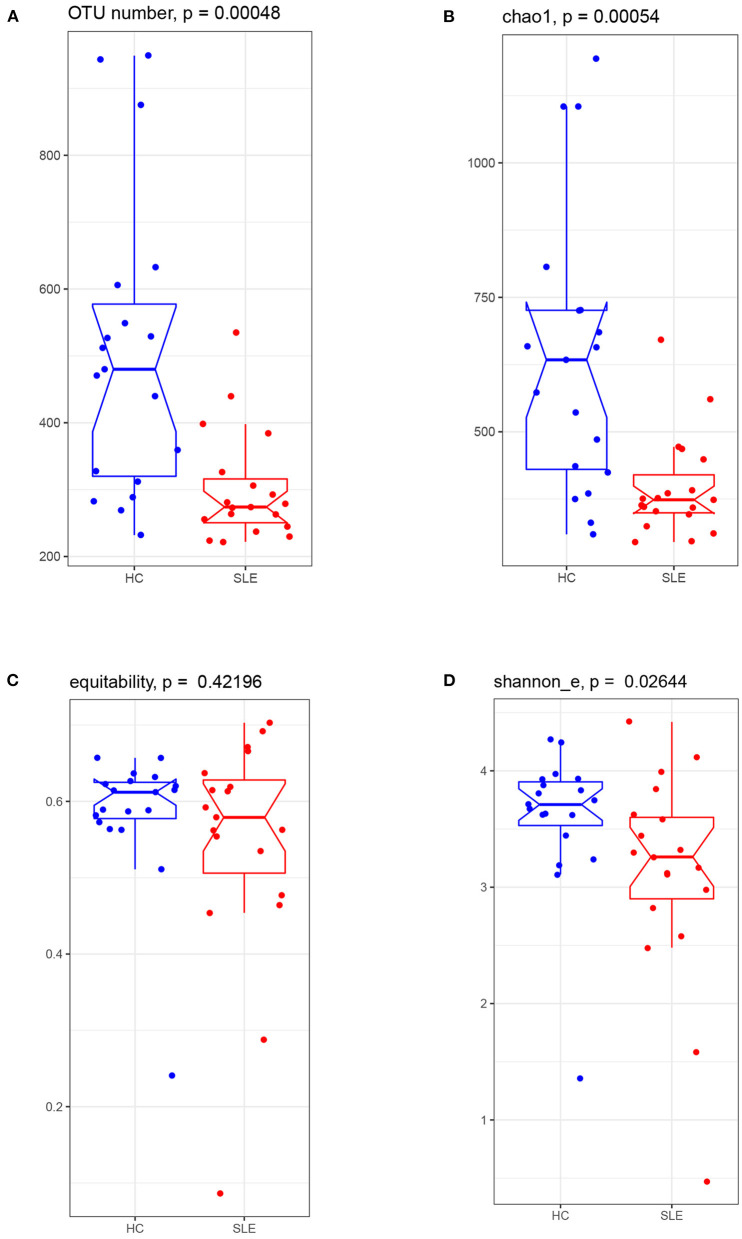
The gut microbiota alpha diversity comparison between the SLE and HC groups. Boxplot visualized five summary statistics (the median, two hinges and two whiskers). Notches represented as median. The lower and upper hinges corresponded to the first and third quartiles (the 25th and 75th percentiles). The whisker extended from the hinge to the largest value no further than 1.5 ^*^ IQR (the distance between the first and third quartiles) from the hinge. Data beyond the end of the whiskers were outlying points. **(A)** observed OTU number, **(B)** richness index of Chao1, **(C)** equability, and **(D)** Shannon_e index.

Sequencing of the 16S rRNA gene yielded 2,485,658 valid reads from 38 successfully sequenced samples. Based on the identity of 0.97, these reads were assigned to 1996 OTUs. Comparison of the microbiota alpha diversity between SLE and HC groups showed lower OTU numbers in the SLE group than in the HC group (274 vs. 480, respectively, with *p* = 0.00048) (Figure 1A). Estimated richness index Chao1 was also lower in SLE group than in HC group (median Chao 1 value of 373.60 vs. 633.80, respectively, with *p* = 0.00054) (Figure 1B). The equitability index of OTUs was comparable (*p* = 0.422) in SLE and HC groups (0.58 vs. 0.61, respectively) (Figure 1C). The Shannon diversity index was also lower (*p* = 0.026) in the SLE group than in the HC group (3.26 vs. 3.71, respectively, Figure 1D). Together, these indices indicate that SLE patients have a lower alpha diversity in their gut microbiota due to a reduced number of OTUs.

### Beta Diversity of the Microbiota in the SLE Group

**Figure 2:**
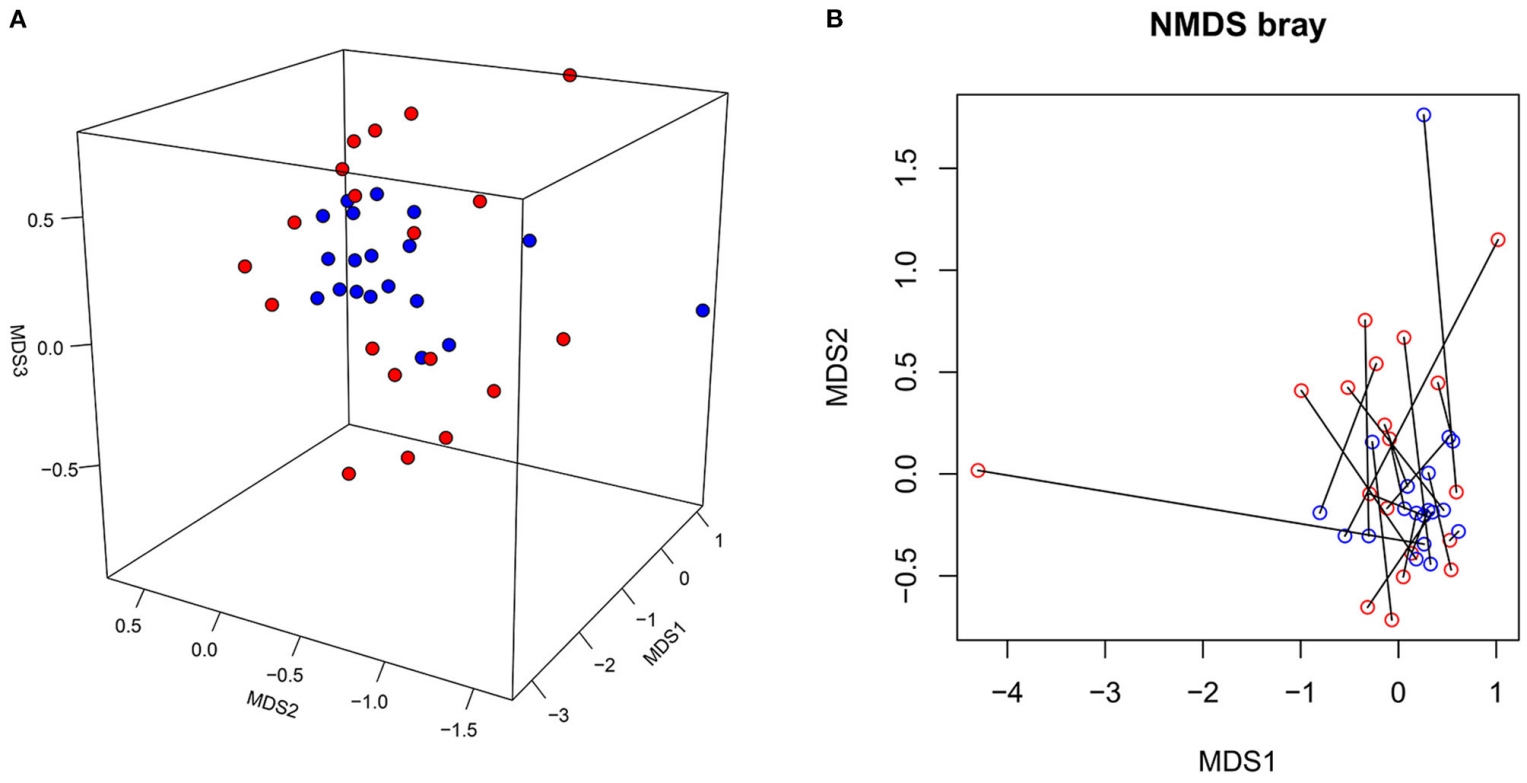
The NMDS plot of gut microbiota from the SLE and HC groups. **(A)** The NMDS coordinates of SLE (red dot) and HC (blue dot) were plotted in 3D review. **(B)** The NMDS coordinates of SLE (red circle) and HC (blue circle) were re-plotted with samples from each disease-control pair connected.

Between-group differences in the beta diversity of gut microbiota were evaluated by filtering the top 20 rich OTUs and performing a Bray distance-based NMDS analysis. This analysis revealed that the microbiota in the SLE group was more dispersed than in the HC group (Figure 2A). By enrolling the top 20 most abundant OTUs, the PERMDISP2 procedure and ANOVA confirmed that the SLE group had a greater heterogeneity of microbiome variance than the HC group (*p* = 0.03798). Re-plotting the NMDS coordinates connected for patient-control pairs showed that these paired coordinates were not located particularly close in the plot (Figure 2B), confirming differences between SLE and HC microbiomes at the community level. An Adonis model including four factors (disease status, sex, age and BMI) revealed that SLE disease status (*p* = 0.003) and age (*p* = 0.027) were significant contributing factors to the differences between SLE and HC microbiomes, but not sex (*p* = 0.900) or BMI (*p* = 0.053). These data indicate that there are differences in the gut microbiota at the community level between SLE and HC groups, and that disease state (SLE vs. healthy) is the most significant contributing factor.

### Taxonomical Changes of the Microbiota in the SLE Group

**Figure 3:**
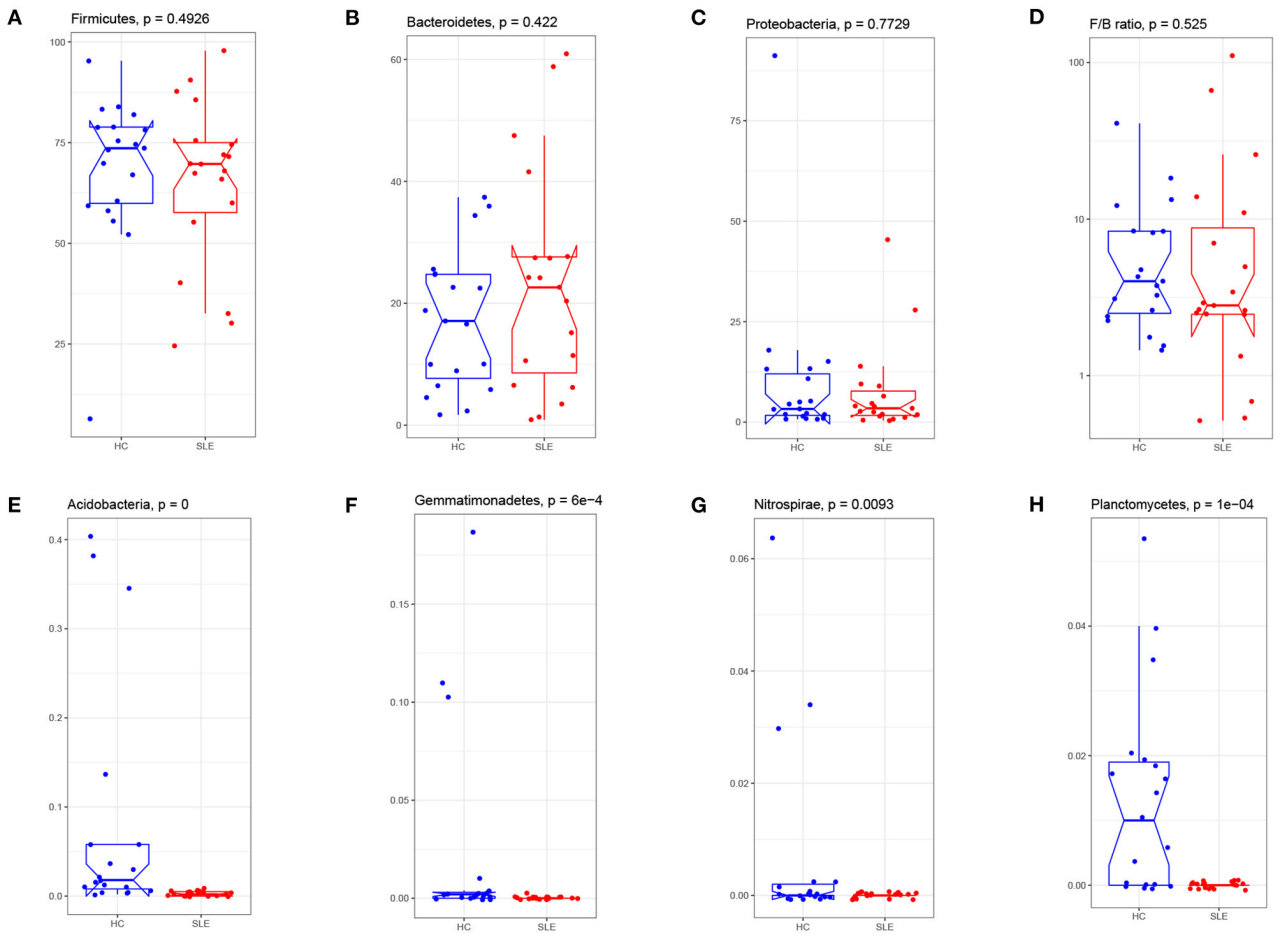
Phylum abundance comparison of gut microbiota between the SLE and HC groups. Three major phyla: the Firmicute **(A)**, Bacteroides **(B)**, Proteobacteria **(C)**, and were compared and the ratio of Firmicute/Bacteroides **(D)** were plotted. The percentage of Acidobacterium **(E)**, Gemmatimonadetes **(F)**, Nitrospirae **(G)**, and Planctomycetes **(H)** in SLE fecal were significantly reduced after FDR adjustment (q < 0.05). F/B: the relative abundance of Firmicute to Bacteroides.

Taxonomic profiling of the SLE group microbiota started at the phylum level. There were no between-group differences for the three major phyla (Firmicutes, Proteobacteria and Bacteroidetes) (Figures 3A–C). The ratio of Firmicutes to Bacteroidetes was lower in the SLE group (Figure 3D), but this was not statistically significant. The percentage of Acidobacteria, Gemmatimonadetes, Nitrospirae and Planctomycetes was remarkably lower in the SLE group (Figures 3E–H). Noted that these four phyla account for a very low proportion of phyla in human gut and there were some extremely high values in the HC group. We reanalyze the abundance of the four phyla after deleting these unnormal values. The results indicated that for Acidobacteria, Gemmatimonadetes and Planctomycetes the distribution differences were still significant between SLE and HC group (*p* = 0.00016, 0.0015, and 0.0009 respectively). For Nitrospirae, the difference was not with statistical significance (*p* = 0.05595).

**Figure 4:**
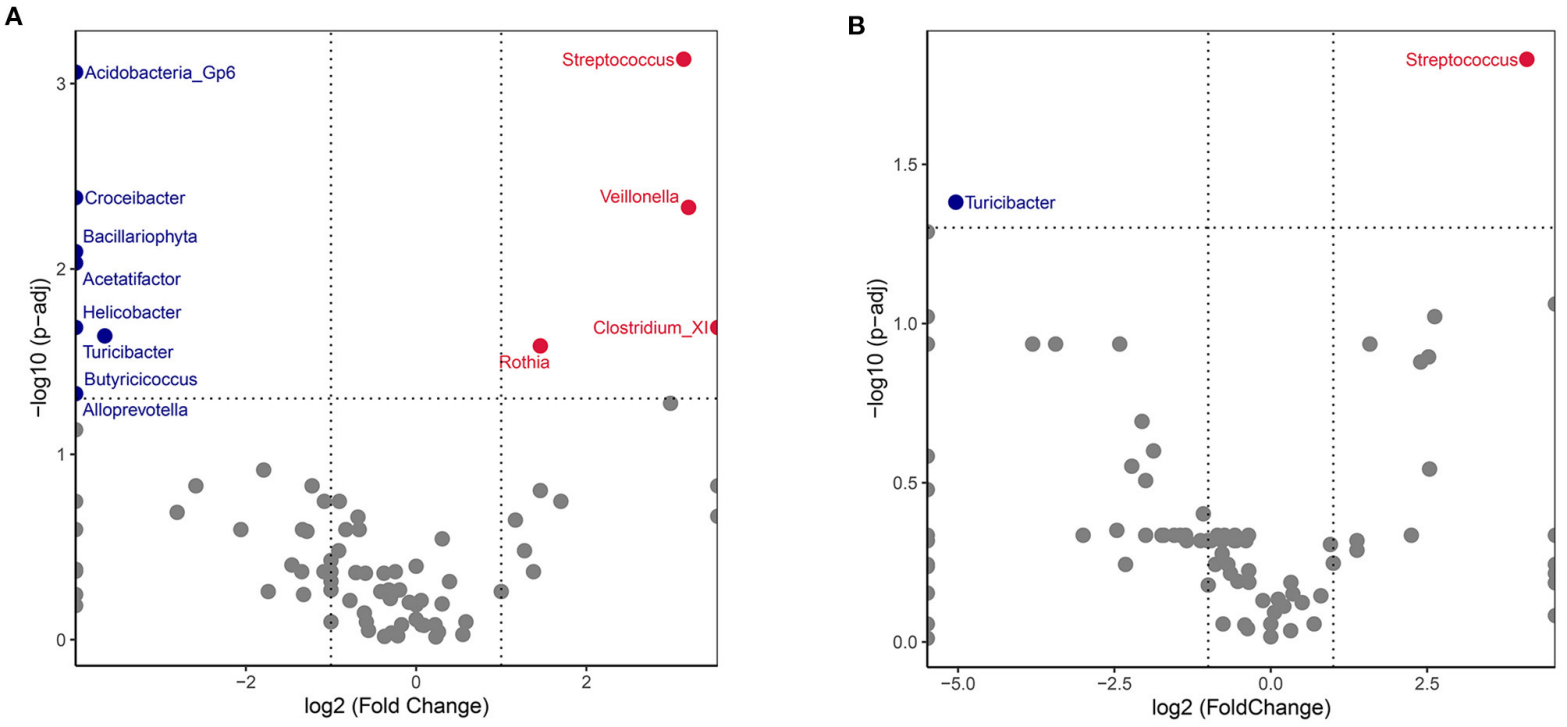
Volcano plot comparing SLE and HC gut microbiota. The genus abundances of all SLE recipients **(A)** or lupus nephritis (LN) subgroup **(B)** were compared with healthy controls. Wilcoxon p-values were calculated and adjusted by the FDR method. Each dot represented a genus. Significantly increased genera of SLE group were plotted and annotated in red, while decreased in blue. The X axis represents the log2 values fold change and the Y axis represents the –log10 value of the adjusted *p*-values.

Between-group comparison of the abundance of genera identified a significant difference for 12 of the 331 genera after FDR adjustment (q < 0.05). Volcano plots for the SLE group showed an increase of *Streptococcus, Veillonella, ClostridiumXI* and *Rothia*, and a decrease in *Acidobacteria_Gp6, Croceibacter, Bacillariophyta, Acetatifactor, Helicobacter, Turicibacter, Butyricicoccus* and *Alloprevotella* (Figure 4A). Patients in the SLE group were segregated based on the presence of the following 4 clinical manifestations: lupus nephritis (LN), rash, arthritis, and blood system involvement. Gut microbiota abundance for each of these subgroups was compared to the control group at the genus level. The abundance of *Streptococcus* was considerably increased in patients with LN, while that of *Turicibacter* was reduced compared to the control group (Figure 4B). The other 3 subgroups (rash, arthritis, and blood involvement) displayed no significant difference for microbiota abundance than control group.

### Network Analysis of the Association Between Microbiome, Diet and SLE

**Figure 5:**
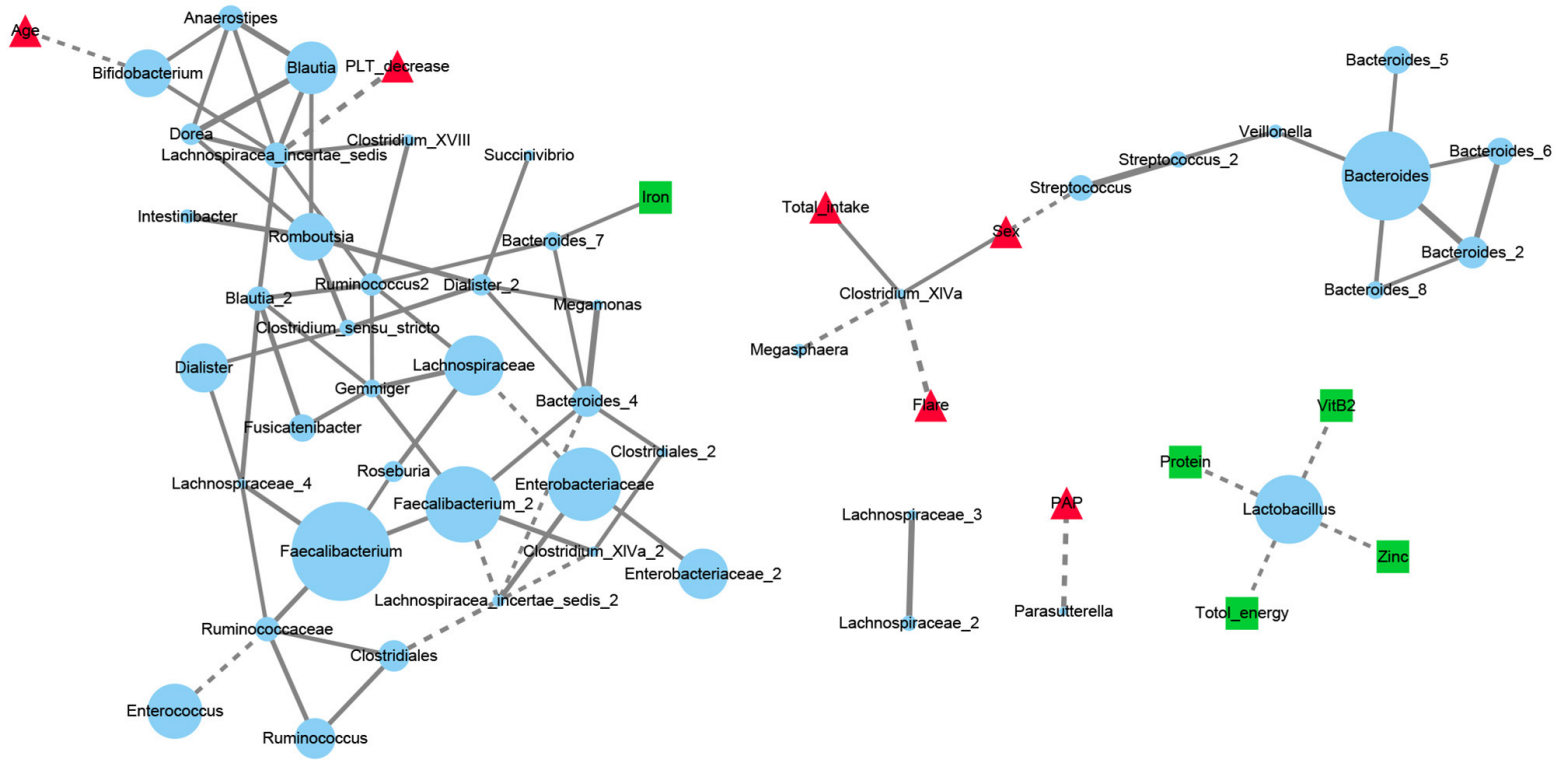
Network analysis of dietary elements, gut microbiota and manifestations of SLE patients. Each blue dot represented for an OTU and its diameter was proportional to the square root of its abundance. Red triangles represented for disease manifestations and green squares for dietary factors. Solid line represented for the significant positive correlations (Spearman r > 0 and FDR adjusted *p* < 0.05) and dashed for negative correlations (Spearman r <0 and FDR adjusted *p* < 0.05). PLT, platelets; Flare: the state of SLE disease flare; PAP, pulmonary artery pressure.

A network analysis was performed to fully describe the covariation between gut microbiota, dietary factors and SLE disease status for SLE group. The interactions with significant correlations (q < 0.05) between microbiota OTUs, 5 dietary elements and 6 SLE clinical manifestations were finally visualized (Figure 5). The most abundant OTUs in the SLE group were two *Faecalibacterium* OTUs located at the center of the microbiota network. They were intertwined with other gut communities but was not directly related to SLE clinical features or to dietary factors. The decrease in blood platelets (PLT) was negatively correlated with an OTU of *Lachnospiracea_incertae_sedis* while the pulmonary artery pressure (PAP) level was negatively correlated with *Parasutterella*. An OTU of *Clostridium_XlVa* showed a distribution difference with sex. It was also positively linked with total amount of daily nutrients intake and negatively with SLE disease flare. *Lactobacillus* OTU that was separate from the core microbiota network negatively correlated with total energy, protein, zinc and vitamin B2 intake.

## Discussion

In the present study, we used 16S rRNA gene sequencing and a FFQ to compare the gut microbiota of patients with SLE and their healthy family members, and to evaluate the association between gut microbiota and dietary patterns. The main findings of our bioinformatic analysis are as follows. First, compared to a healthy control group of family members, thus controlling for living situation and diet, the fecal microbiota in patients with SLE was characterized by a lower richness of alpha diversity and higher heterogeneity as well as an abundance of *Streptococcus*. Differences in microbiome between the two groups were mainly attributed to SLE disease status and age. Network analysis revealed that a *Lactobacillus OTU* mainly covaried with dietary factors, whereas *Clostridium_XlVa, Lachnospiracea_incertae_sedis* and *Parasutterella* OTUs mainly covaried with clinical features of SLE.

OTUs and Chao1 index were indicative of a decrease in the alpha diversity of SLE bacterial communities. This is consistent with previous studies which reported that Chao1 estimates of total OTUs were significantly lower in SLE patients, tending toward an inverse correlation between Chao1 and SLEDAI (19). Similarly, our finding of significant decrease in the Shannon index for SLE is consistent with a study of untreated Chinese SLE patients (20). With regards to the beta diversity of the gut microbiome, the NMDS analysis showed more heterogeneity for the SLE group than for the HC group. This has also been observed for other inflammatory diseases, such as IBD, Crohn's disease (CD) and asthma (21–23). The relationship between the disturbance imbalance of gut microbiota and immune diseases remains to be fully clarified. One theory is that gut microbiota antigens play an important role in the differentiation and maturation of T and B cells in humans after birth. Thus, bacterial dysbiosis may lead to impaired immune tolerance and increased susceptibility to immune disorders (24). In a murine model, a reduced gut microbiota diversity following antibiotic treatment was associated to gut inflammation (25). Moreover, according to the “hygiene hypothesis” that the absence of certain microbes and lower exposure to bacterial antigen leads to the rise of allergies and autoimmune disorder, the relatively high prevalence of SLE in developed countries is partly due to the reduced richness of microbes (26, 27).

At the phylum level, Firmicutes, Bacteroidetes and Proteobacteria are the three main bacterial phyla in the human intestinal tract. The observed decrease in the Firmicutes to Bacteroidetes ratio in fecal samples of SLE patients has been also reported in some studies, but not in others (5, 10, 28). We note that the decrease in Firmicutes and Firmicutes to Bacteroidetes ratio was not found to be significant. In contrast, Acidobacteria, Gemmatimonadetes, Nitrospirae and Planctomycetes phyla were significantly reduced in the SLE group, although they account for a very low proportion of phyla in the human gut. They are mainly abundant in soil and related to diverse metabolic pathways, such as carbon metabolism and oxygen utilization (29, 30). The reports of their relationship with human diseases were relative less. Acidobacteria was remarkably decreased in idiopathic nephrotic syndrome (31) and Gemmatimonadetes was found reduced in osteopenia patients than osteoporosis patients (32). In-depth researches about the four bacteria phyla in autoimmune diseases are required.

At the genus level, SLE and HC groups gut microbiota were clearly different, with SLE showing an increase in *Streptococcus, Veillonella, Clostridium XI* and *Rothia*. Among these, *Streptococcus* has attracted significant attention, with levels being particularly high among LN patients. A previous study reported an enrichment of *Streptococcus* and *Veillonella*, positively associated with SLEDAI, in the gut of SLE patients (33). Since *Streptococcus* is the most predominant commensal and common opportunistic infection-causing bacteria in humans, its excess may affect the relationship between microflora and SLE pathogenesis. First, bacterial infection can augment the autoimmune response: *Streptococcus* combined with *Veillonella* isolated from the human small intestine microbiota inhibited IL-12p70 production and augmented IL-8, IL-6, IL-10 and TNF-α responses (34). Moreover, via a “molecular mimicry” mechanism, some species of *Streptococcus* use antigen presentation to induce the initial activation of B cells and specific CD4^+^ T cells. Indeed, antibodies generated against bacterial antigens can be cross-reactive to the host tissue (35, 36). The anti-dsDNA antibody in SLE share a common epitope of a pentapeptide with a polysaccharide of *Streptococcus pneumoniae* (37). Except for Streptococcus, another gram-positive bacterium Ruminococcus gnavus (RG) was reported expanded in SLE by Silverman (19). High levels of anti-RG strain-restricted antibodies were detected in SLE patients with active nephritis. The lipoglycans of cell wall of RG was confirmed with antigenic properties to trigger immune response in SLE. In our cohort, we did not find a special expression of *RG* while Li et al. reported a decrease of RG in active SLE (SLEDAI>8) (33). This difference may be caused by the disparity of subjects' regions, races and disease activity as well as the sample sizes of cohorts. It is also important to known that multiple pathobionts, singly or in combination, may contribute to SLE pathogenesis (19). Second, *Streptococcus* is enriched in the oral cavity and upper intestinal tract in healthy humans; its overgrowth in the lower intestine in SLE patients suggests flora relocation in SLE (38, 39). Several studies have reported a higher abundance of fecal *Streptococcus* associated with liver cirrhosis and liver failure (40, 41). Since *Streptococcus* and its subsidiary products can interfere with the mucosal immune system, research on the extensive mechanism of Streptococcal translocation in SLE is warranted (42, 43).

The relationship between dietary factors and disease has long been a topic of research interest. Our use of a semi-quantitative FFQ revealed that there were no significant differences in main daily energy and nutrients intake between SLE and HC groups. However, network analysis revealed complex connections among the gut microbiota OTUs, diet, and the clinical manifestations of SLE. Among them, genus *Lactobacillus* and *Clostridium_XlVa* were studied a lot in autoimmune diseases. Lactobacillus, whose OTU was negatively correlated with total energy, protein, zinc, and VitB2 intake in our research, has a reported beneficial probiotic effect in ameliorating lupus symptoms and autoantibody production (44, 45). However, Zegarra-Ruiz et al. showed that *Lactobacillus* has opposite pathogenic effects in an SLE mice model (46). In our study, an OTU of *Lactobacillus* was linked with dietary elements and isolated from the core flora, suggesting that it is seldom affected by other gut microbes. Therefore, SLE treatment based on dietary supplementation or depletion of *Lactobacillus* may be both feasible and controllable. An OTU of *Clostridium_XlVa* was noted with a negative correlation with SLE disease flare. It was reported that clusters IV and XIVa of the genus *Clostridium* promoted T regulatory cells (Tregs) accumulation (47). Since Tregs quantity and function impairment has been established with SLE pathogenesis (48, 49), the role of *Clostridium_XlVa* in the immune system of SLE need to be noticed.

Finally, our study has some limitations. First, the sample size used was small, even though it only included inpatient with SLE. Also, most patients in the SLE group were female, which is expected from the sex distribution of this disease, but nearly half of their accompanying family members in the control group were their male husbands, therefore the sex distribution between the two groups is not comparable. To account for this sex bias, we used a multi-factor ANOVA with an Adonis model which showed that sex was not a contributing factor to the observed differences in the microbiota between the two groups. we also did a correlation analysis of sex with other variables (50 top abundant microbiota OTUs and 50 dietary and clinical factors) in SLE and HC group. It showed that sex was only associated with total nutrient intake. The data was listed in the Supplementary Data S1. Nevertheless, larger samples are needed in future studies to confirm this finding.

## Conclusion

Collectively, our comparison of gut microbiota between SLE patients and their healthy family members showed a specific alteration in the SLE group gut microbiome. Also, our findings confirm an association between gut microbiota, dietary intake and SLE clinical manifestations. Further research toward elucidating the precise interactions between microbiota, diet and host immune system may offer novel intervention targets for SLE treatment.

## Data Availability Statement

The datasets presented in this study can be found in online repositories. The names of the repository/repositories and accession number can be found at NCBI Short Reads Archive, accession number: PRJNA648296.

## Ethics Statement

The studies involving human participants were reviewed and approved by the Ethics Committee of Qilu Hospital of Shandong University. The patients/participants provided their written informed consent to participate in this study.

## Author Contributions

ML and XW conceived the study and performed most of the experiments. ML analyzed the data. XW wrote the manuscript. LS and QS helped enroll patients and revised the manuscript. QL and XQ helped with the experiments. All authors contributed to the article and approved the submitted version.

## Funding

This work was funded by the National Natural Science Foundation for Young Scientist of China: 81701605 and 81700457.

## Conflict of Interest

The authors declare that the research was conducted in the absence of any commercial or financial relationships that could be construed as a potential conflict of interest.

## Publisher's Note

All claims expressed in this article are solely those of the authors and do not necessarily represent those of their affiliated organizations, or those of the publisher, the editors and the reviewers. Any product that may be evaluated in this article, or claim that may be made by its manufacturer, is not guaranteed or endorsed by the publisher.

## Supplementary Material

Supplementary Data S1 The spearman correlation analysis of sex with other variables for SLE and HCs was list in the datasheet.
